# Influence of Deposition Parameters of TiO_2_ + CuO Coating on the Membranes Surface Used in the Filtration Process of Dairy Wastewater on Their Functional Properties

**DOI:** 10.3390/membranes11040290

**Published:** 2021-04-16

**Authors:** Joanna Kacprzyńska-Gołacka, Monika Łożyńska, Wioletta Barszcz, Sylwia Sowa, Piotr Wieciński, Ewa Woskowicz, Maciej Życki

**Affiliations:** 1 Łukasiewicz Research Network—Institute for Sustainable Technology, 6/10 Pułaskiego St., 26-600 Radom, Poland; monika.lozynska@itee.lukasiewicz.gov.pl (M.Ł.); wioletta.barszcz@itee.lukasiewicz.gov.pl (W.B.); sylwia.sowa@itee.lukasiewicz.gov.pl (S.S.); ewa.woskowicz@itee.lukasiewicz.gov.pl (E.W.); maciej.zycki@itee.lukasiewicz.gov.pl (M.Ż.); 2 Faculty of Chemistry, Warsaw University of Technology, 3 Noakowskiego St., 00-664 Warsaw, Poland; piotr.wiecinski@gmail.com

**Keywords:** polyamide membranes, MS-PVD method, TiO_2_ + CuO coatings, bactericidal and hydrophilic properties

## Abstract

A novel approach of the deposition of two-component coating consisting of TiO_2_ and CuO on polymer membranes by MS-PVD method was presented in this work. This confirmed the possibility of using thin functional coatings for the modification of polymer membranes. The influence of technological parameters of the coating deposition on the membrane’s structure, chemical composition and functional properties (hydrophilic, photocatalytic and bactericidal properties) were analyzed using SEM. Model microorganism such as *Escherichia coli* and *Bacillus subtilis* have been used to check the antibacterial properties. The results indicated that doping with CuO highlights the potential of bactericidal efficiency. The surface properties of the membranes were evaluated with the surface free energy. For evaluating photocatalytic properties, the UV and visible light were used. The filtration tests showed that polymer membranes treated with two-component TiO_2_ + CuO coatings have a permeate flux similar to the reference material (non-coated membrane). The obtained results constitute a very promising perspective of the potential application of magnetron sputtering for deposition of TiO_2_ + CuO coatings in the prevention of biofouling resulted from the membrane filtration of dairy wastewater.

## 1. Introduction

Polymer membranes play an increasingly important role in various filtration processes. They are widely used in filtration processes of technological wastewater in various types of the industry thanks to such advantages as simple application, efficiency, and low cost [1,2,3]. The cost associated with the operating process of the membranes is a significant problem for using membrane filtration. Currently, a lot of filtration processes e.g., in the dairy industry, are concerning the filtration of complex mixtures containing substances with different nature and activity, which can be deposited on the surface and in the pores of the membrane, reducing its filtration properties [4]. It is leading to lower efficiency and higher filtration cost. In this case, cleaning and more frequently replacing the membranes is a necessity. 

The conducted analysis of knowledge in this topic allowed us to select the main problems in the process of membrane filtration for dairy wastewater. Milk is a very complicated substance for the filtration process, mainly due to the wide range of particle sizes (1 nm–20 µm), the high concentration of dispersed ingredients (13 wt. %), as well as their diversity. The composition of milk is based on somatic cells (15 nm–6 µm), microdroplets of fat (15 nm–0.2 µm), bacteria (6 nm–0.2 µm) and casein micelles (0.3 nm–0.03 µm) [5,6]. During the filtration process, a strong decrease occurs in the flux, which is caused by the deposition of impurities from the filtered medium (fouling) on the membrane’s surface [4]. Membrane destruction can also occur as a result of the deposition of microorganisms on the surface intensified by the growth of biomass. The specificity of the dairy wastewater filtration process can create good conditions for the development of biofilm [7]. It leads to a significant reduction in the efficiency and increase in filtration process cost and can lead to the colonization of working surfaces and elements of industrial installations with microbial metabolism products generating a high risk of contamination microbial products [8,9]. Therefore increasingly, filtration membranes used in the food industry should also fulfill additional functions. Filtration membranes should be characterized by surface properties that make it impossible to bind and build-up the potential impurities, which leads to the deterioration of the membrane’s filtration parameters. The bactericidal properties allow reducing the intensity of biofilm formation on the surface and the risk of biological contamination of the filtered medium. In addition, the catalytic properties of the membrane can be useful for the process, which is carried out by accelerating the chemical transformations, enhancing the decomposition and transformation of substances in the system causing the neutralization of impurities from the filtration process [10]. 

One of the most promising ways to enrich the functional properties of polymer membranes is surface modification [11,12,13,14,15,16,17,18,19,20]. So far, many attempts have been made to modify the surface of the membranes that can help to control membrane contamination. It is still a problem to modify the membranes’ surface without disturbing its good separation and filtration performance during filtration carried out under various operating conditions [21,22]. It is possible to give bactericidal properties to the polymer membrane by applying a thin bactericidal layer on its surface using the magnetron sputtering method (MS-PVD). CuO-based coating is such as type of materials. However, these coatings are characterized by a very high contact angle. It affects the flow of the filtered medium through the membrane and thus reduces the speed of the filtration process. According to the authors, in order to improve the bactericidal properties of polymer membranes, a multi-component coating should be used, consisting of TiO_2_, ensuring good hydrophilic properties and CuO, ensuring appropriate bactericidal properties.

The aim of the work was to develop appropriate technological parameters for plasma deposition of titanium oxide and copper oxide (TiO_2_ + CuO). It made it possible for the generation of stable coating on the surface of filtration materials with good separation capability, high permeability, and antibacterial properties. In this study, all of the modified membranes were tested in terms of the structure of the deposited layers, photocatalytic and hydrophilic properties, and antibacterial properties toward two representative bacterial groups, Gram-positive (*Bacillus subtilis*), and Gram-negative (*Escherichia coli*). The effectiveness of the modified membranes under operating conditions was also confirmed. We examined their filtration and separation properties during tests with the model dairy wastewater.

## 2. Materials and Methods 

### 2.1. MS-PVD Process

For surface modification of flat sheet polyamide membranes (0.22 µm, GVS) the MS-PVD process was used. The samples were subjected to the magnetron sputtering method using a Standard 3 device made by Łukasiewicz Research Networks-Institute for Sustainable Technology (Radom, Poland). The equipment has two independent plasma sources located on the same wall of the chamber with a common deposition zone. For the deposition process two metallic targets, Ti (99.99% purity) and Cu (99.99% purity) with 100 mm diameter, were used. The distance of the plasma source from the sample was 200 mm. The membranes were coated at room temperature with a reactive gas atmosphere with oxygen and argon mixture. The coatings were produced using different magnetron power sources without the substrate polarization. The detailed parameters of the deposition process are presented in Table 1.

### 2.2. Surface Structure and Chemical Composition of the Coatings

The scanning electron microscopy Hitachi Su-8000 (SEM; Tokyo, Japan) equipped with an electron gun with cold field emission was used to investigate the produced TiO_2_ + CuO coatings. As far as membranes are sensitive material this type of electron source provides very good resolution with a relatively low beam current. For observation, the secondary electron signal (SE) was used. An additional conductive layer was not necessary to apply to the membranes. The EDS method was used for the determination of the chemical composition of deposited two-component (TiO_2_ + CuO) coatings, which included quantitative atomic analysis of titanium, copper and oxygen.

### 2.3. Antibacterial Test

The antibacterial properties for all coatings surface were investigated versus Gram-negative bacteria (*Escherichia coli*) and Gram-positive bacteria (*Bacillus subtilis*) by colony counting method. The membranes were sterilized with UV-C in laminar cabinet for 30 min before the microbiological tests. For the preparation of inoculum the subculture from the slant was suspended in 20 cm^3^ of sterile Luria broth (LB Agar Miller, VWR Chemical) culture medium, and then shaken at 2.21 Hz at 37 °C for 24 h. For achieving a countable number of colonies on the membranes it was necessary to dilute the obtained suspension by serial dilution method. In the next step from the prepared dilution were taken 0.04 cm^3^ (for *Escherichia coli*) and 0.1 cm^3^ (for *Bacillus subtilis*) of suspension and transferred to a sterile 1000 cm^3^ phosphate buffer. After that, 10 cm^3^ of the suspension, which was prepared in this way was filtered through membranes under a pressure of 0.05 bar and placed on Luria broth (LB) with agar plates and incubated at 37 °C for 24 h. The bacterial colonies, which had grown on the membranes were counted after this time (CFU). The reference sample in the research was non-coated membrane. The results obtained for membranes with deposited TiO_2_ + CuO coatings were expressed as the percentage (%) reduction in bacterial cell viability in relation to the non-coated membrane. All experiments were carried out three times.

### 2.4. Photocatalytic Properties

The photocatalytic properties of the membranes were based on an analysis of the degree of methylene blue degradation (0.1% *v/v*) under the UV light and visible light. The non-coated and coated membranes with TiO_2_ + CuO were placed in Petri dishes. The dye solution with 20 cm^3^ volume was transferred to the surface of the membrane. UV-A lamp and daylight, were used for the irradiation experiment. The spectrophotometric measurements were made at the wavelength of 665 nm using a Hach DR 6000 spectrophotometer after 24 h exposure to UV and 8, 24, 48, and 72 h exposure to daylight. The tests were repeated three times for each membrane sample. The reference test was a dye after contact with the non-coated membrane to take into account the potential effect of the dye on the membrane.

### 2.5. Surface Free Energy

The surface free energy (SFE) of two-component tested TiO_2_ + CuO coatings was determined by the Owens-Wendt method based on the measurements of contact angles of polar liquid (deionized water) and non-polar liquid (diodomethane), according to Equations (1)–(3).

(1) $$\gamma_{M}=\gamma_{M}^{d}+\gamma_{M}^{p}$$

(2) $$\gamma_{ML}=\gamma_{M}+\gamma_{L}-2\left(\sqrt{\left(\gamma_{M}^{d}\gamma_{L}^{d}\right)}+\sqrt{\gamma_{M}^{p}\gamma_{L}^{p})}\right)$$

(3) $$\frac{\gamma_{L}\left(1+cos\theta \right)}{2}=\sqrt{\gamma_{M}^{d}\gamma_{L}^{d}}-2\sqrt{\gamma_{M}^{p}\gamma_{L}^{p}}$$

where: $\gamma_{M}-\;\mathrm{SFE}\;\mathrm{of}\;\mathrm{the}\;\mathrm{membrane};\;\gamma^{p}$—polar component; $\gamma^{d}$—dispersion component; $\gamma_{ML}$—SFE on the contact surface between membrane and test liquid; $\gamma_{L}$—SFE of test liquid, θ—contact angle between tested surface of the membrane and standard liquid.

The contact angles were measured using the sessile drop method (drop volume—2 µL). The goniometer was used for this purpose, which was manufactured at Łukasiewicz-ITeE (Radom, Poland). The measurement method is based on the drop shape analysis according to Young’s equation [23]. Using computer image processing allowed to increase the speed, accuracy, and precision of the used methods. The computer software is based on the numerical solution of the Laplace-Young equation for the capillary:

(4) $$\Delta \rho =\sigma \left(\frac{1}{R_{1}}+\frac{1}{R_{2}}\right)$$

A digital camera measures the parameters of the drop (diameter, height, etc.). In the case of sessile drop, parameters depend on the angle which forms drop with the surface. The obtained results are compared with the so-called dimensionless (theoretical) profiles, which are solutions of the Laplace-Young equation. In this method the surface or interfacial tension is determined from the formula:

(5) $$\sigma =\frac{\Delta \rho gR_{o}^{2}}{\beta }$$

where, *R_o_*—radius of curvature of the drop at the top, *β*—drop shape parameter, ∆*ρ*—difference between density of drop and environment, *g*—standard gravity.

In order to assess the wettability of the coatings, solid polyamide samples with all types of coatings were used for testing. Ten measurements were made for each coating. Coarse errors were rejected based on the performed Dixon tests. Averaged values of contact angles for deionized water and diodomethane were used to calculate the surface free energy of each tested coating. The standard deviation of the surface free energy was determined according to the error propagation method.

### 2.6. Filtration and Separation Performance

The membranes modified by TiO_2_ + CuO coatings were tested in terms of filtration and separation properties. The non-coated membrane was used as a reference. The filtration performance was assessed by analyzing the volumetric permeate flux, which was calculated from the time needed to filter 100 cm^3^ of deionized water through an 8 cm^2^ membrane at a pressure of 0.5 bar. 

The separation properties of membranes were examined using model dairy wastewater filtered through the cross-flow membrane module (Sterlitech). This module enables the process to be carried out with continuous concentration of the feed due to returning the retentate to the feed tank. The process was carried out at a pressure of 1 bar until the feed stream was concentrated four times. After two-fold (VRF 2) and four-fold (VRF 4) concentrations of the stream, samples of the feed, permeate, and retentate were taken and the turbidity, chemical oxygen demand (COD), total bound nitrogen (TNb), total phosphorus (TP) levels were determined.

## 3. Results and Discussion

### 3.1. Structure and Elemental Composition Characterization

**Figure 1:**
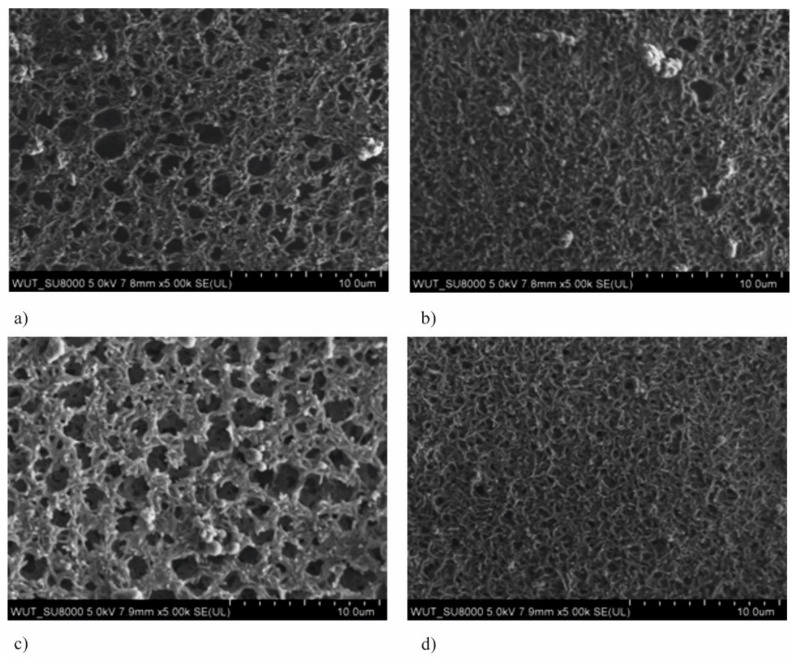
SEM images of the membranes with TiO_2_ + CuO coatings deposited at different magnetron powers PM in two characteristic areas with different pore sizes, (**a**) P_M-Cu_ = 15 W, P_M-Ti_ = 650 W, t = 30 s, area with a high proportion of large spherical pores, (**b**) P_M-Cu_ = 15 W, P_M-Ti_ = 650 W, t = 30 s, area with a high proportion of small irregular pores (**c**) P_M-Cu_ = 15 W, P_M-Ti_ = 1000 W, t = 30 s area with a high proportion of large spherical pores, (**d**) P_M-Cu_ = 15 W, P_M-Ti_ = 1000 W, t = 30 s area with a high proportion of small irregular pores.

**Figure 2:**
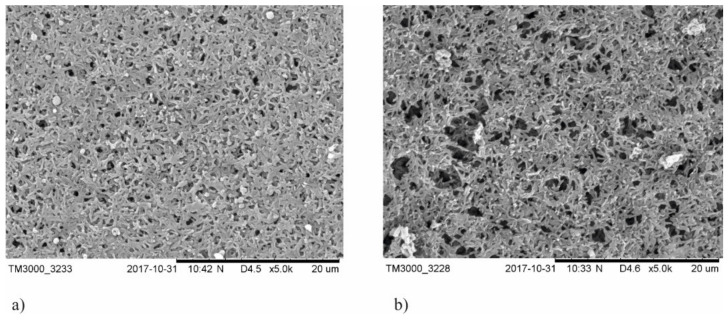
SEM images of the non-coated membranes in two characteristic areas with different pores sizes: (**a**) area with a high proportion of small irregular pores; (**b**) area with a high proportion of large spherical pores.

**Figure 3:**
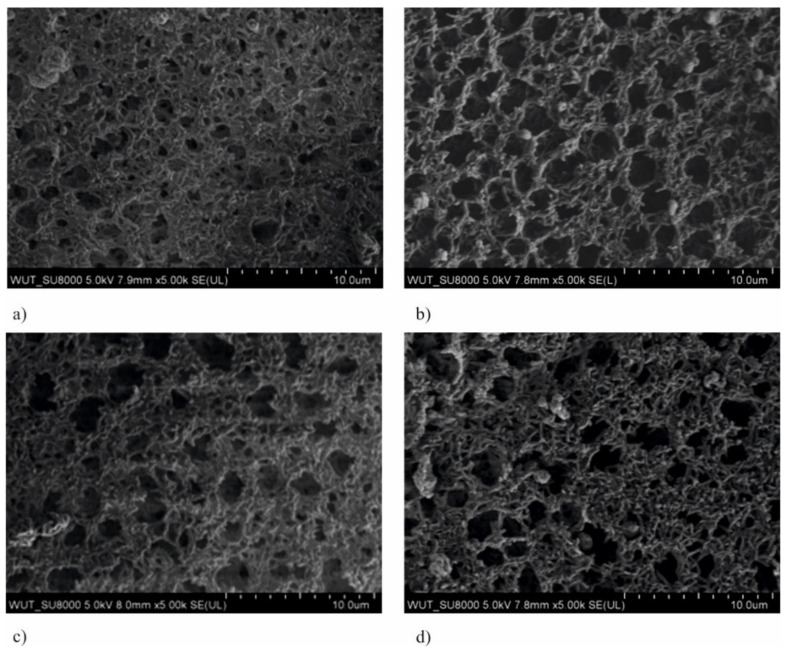
SEM images of the membranes with TiO_2_ + CuO coatings deposited at different magnetron powers P_M_ (**a**) P_M-Cu_ = 100 W, P_M-Ti_ = 650 W, t = 30 s, (**b**) P_M-Cu_ = 200 W, P_M-Ti_ = 650 W, t = 30 s, (**c**) P_M-Cu_ = 100 W, P_M-Ti_ = 1000 W, t = 30 s, (**d**) P_M-Cu_ = 200 W, P_M-Ti_ = 1000 W, t = 30 s.

SEM observations of polyamide membranes with TiO_2_ + CuO coatings are shown in Figure 1 and Figure 2. In the case of membranes covered with coatings deposited at magnetron power P_M-Cu_ = 15 W, two areas of different porosity (Figure 1) characteristic for non-coated membranes (Figure 2) were observed. One of the observed areas was characterized by a high proportion of large pores with approximate size 2 μm and almost spherical shape (Figure 1a,c, and Figure 2a). In the second area, there were smaller pores (500 nm) of irregular shape, while there were significantly fewer large spherical pores (Figure 1b,d, and Figure 2b). This indicates that the deposition process of the TiO_2_ + CuO coating with magnetron power P_M-Cu_ = 15 W did not disturb the porous structure of the membrane. The magnetron power of copper above 100 W (P_M-Cu_ = 100 W or P_M-Cu_ = 200 W) resulted in an increase in the diameter of the spherical pores and their share in the membrane structure with the simultaneous disappearance of areas with smaller pores (500 nm) of irregular shape (Figure 3). The observed changes can have an adverse effect on the filtration properties of the membrane. The influence of the Ti magnetron power (P_M-Ti_) in the range 650–1000 W was not observed on changes in membrane morphology.

**Figure 4:**
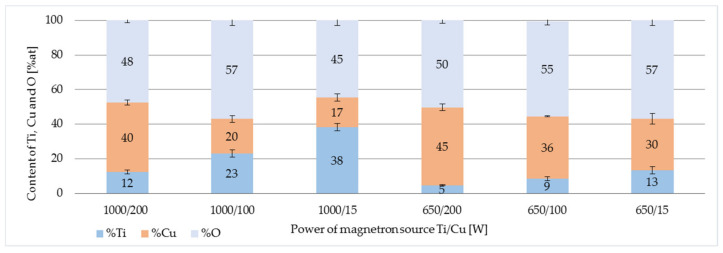
Elemental composition of TiO_2_ + CuO coatings deposited at different power of magnetron source.

The analysis of the elemental composition involved the assessment of the magnetron power effect on the percentage share of individual metallic elements in the chemical composition of the coating which included quantitative atomic analysis of titanium, copper and oxygen. The analysis also showed that the share of carbon coming from the base material. However, the quantitative analysis of carbon by EDS technique is fraught with errors. Only three items were quantified on the software. With such settings, the system automatically sums up all analyzed elements up to 100%. The obtained results showed that the TiO_2_ + CuO coatings (Figure 4) deposited at higher magnetron power P_M-Ti_ = 1000 W were characterized by a higher proportion of Ti compared to the coatings produced at the magnetron power of P_M-Ti_ = 650 W. Increasing the magnetron power of copper (P_M-Cu_) in the deposition process increased the share of copper in the chemical composition of the tested coatings.

### 3.2. Antibacterial Test

**Figure 5:**
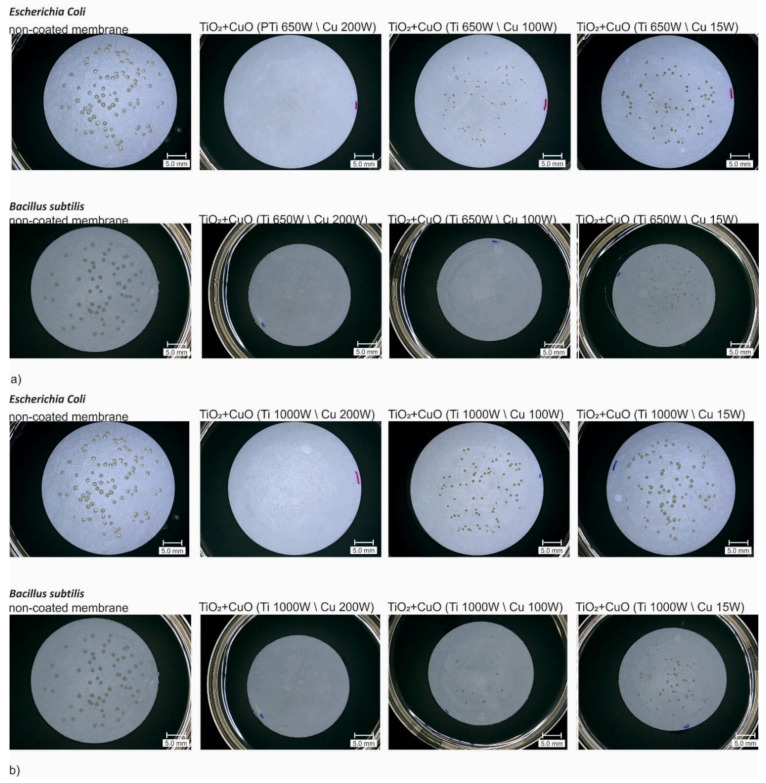
Comparison of 3D microscope images of non-coated membranes and membranes covered with TiO_2_ + CuO coatings after vacuum filtration of the bacterial suspension: (**a**) P_M-Ti_ = 650 W; P_M-Cu_ = 200 W, 100 W, 15 W; (**b**) P_M-Ti_ = 1000 W; P_M-Cu_ = 200 W, 100 W, 15 W.

The antimicrobial activity of TiO_2_ + CuO coatings deposited on polymer membranes was determined against *Bacillus subtilis* and *Escherichia coli*. The microscope images of membranes covered with two-component TiO_2_ + CuO coatings after filtering of bacterial suspension are shown in Figure 5. The microscopic observations showed significant differences in the process of bacterial growth on the surface of membranes covered with two-component TiO_2_ + CuO coatings compared to non-coated membranes.

**Figure 6:**
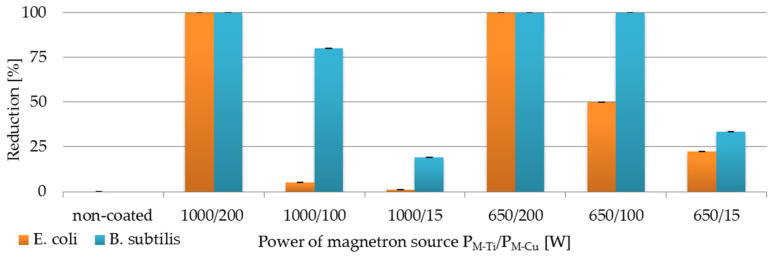
Reduction (%) in viability of *E.coli* and *B. subtilis* on the non-coated membrane and membranes covered with TiO_2_ + CuO coatings deposited within 30 s at different magnetron powers (P_M-Ti_/P_M-Cu_).

The antibacterial activity of each membrane coated with TiO_2_ + CuO was presented in Figure 6. As can be seen in Figure 6, bactericidal activity was different for all modified membranes. The tested coatings showed stronger bactericidal activity towards *Bacillus subtilis* than *Escherichia coli*. It is probably related to the structure of the outer layers of bacterial cells. The coatings with copper in a structure have higher efficiency of inhibiting pathogens growth which is related to mechanisms of action of Cu [24,25]. Copper compounds lead to the death of the bacterial cell as a result of the generated radicals, which penetrate the envelope accumulating inside the cell and disturbing the metabolic processes [25]. The presented results indicated that the highest antibacterial activity against both bacteria was characterized by the TiO_2_ + CuO coatings deposited at the power of magnetron sources: P_M-Ti_ = 1000 W, P_M-Cu_ = 200 W, and P_M-Ti_ = 650 W, P_M-Cu_ = 200 W. These coatings resulted in a complete reduction in bacterial colony counts (CFU).

The demonstrated results indicate that the antimicrobial activity of the coatings depends on the elemental composition. Increasing the magnetron power of copper (P_M-Cu_) can increase the amount of copper in the coatings as was shown in Figure 4. The tested composite coatings with a higher content of titanium showed a much lower antibacterial activity. Figure 6 presents TiO_2_ + CuO coatings, which contain more amount of titanium (Ti) than copper (Cu). This influenced on reduction in the number of bacterial colonies of only 1–20% and 19–33% for *E. coli* and *B. subtilis*, respectively. Researchers by Phan D.N. et al. found that as the copper content of the composite increased, the antibacterial properties were higher [26].

### 3.3. Photocatalytic Properties

**Figure 7:**
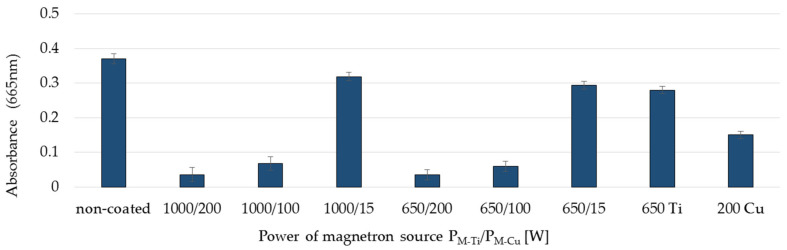
Comparison of the absorbance after exposure of the coatings to UV light for 24 h for non-coated membrane and membranes covered with TiO_2_ + CuO, Ti and Cu coatings deposited within 30 s at different magnetron power (P_M-Ti_/P_M-Cu_).

Based on the obtained results, it can be concluded that the factor determining the photocatalytic properties is the concentration of copper in the tested coating (Figure 7). The highest photocatalytic effects were observed for the coatings deposited at the magnetron powers (P_M-Cu_) 100 W and 200 W. All these coatings allowed to reduce the dye by about 90% after 24 h. Doping of titanium dioxide (TiO_2_) coatings with copper oxide (CuO) showed a synergistic effect on the photocatalytic properties compared to the one-component coatings such as Ti and Cu. This effect is the result of the Cu inclusion in the structure of TiO_2_, which contributes to the increased activity of the photocatalytic properties and the production of more free radicals [27,28,29].

**Figure 8:**
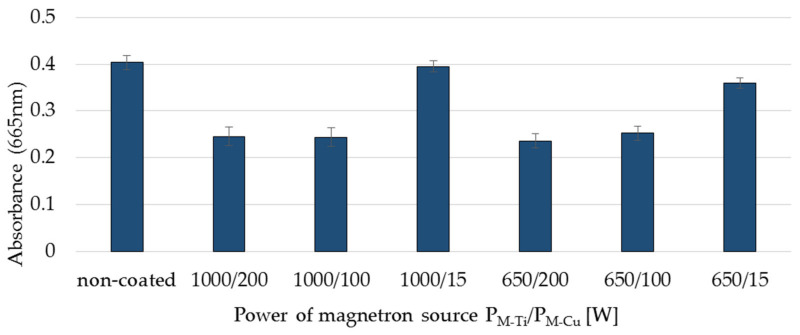
Comparison of the absorbance after exposure of the coatings to daylight for 24 h for non-coated membrane and membranes covered with TiO_2_ + CuO coatings deposited within 30 s at different magnetron power (P_M-Ti_/P_M-Cu_).

**Figure 9:**
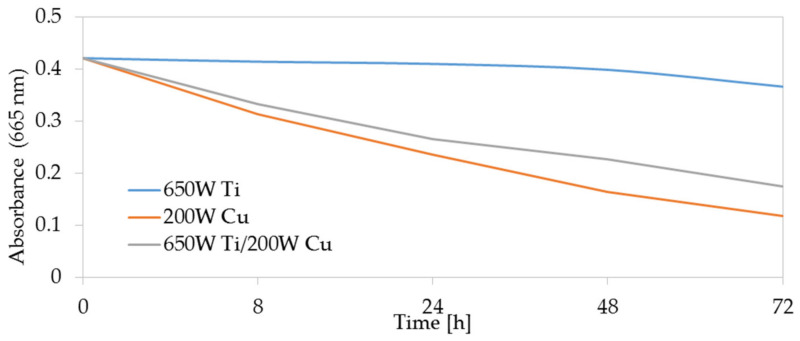
Comparison of absorbance after exposure of single-component Ti and Cu coatings and two-component TiO_2_ + CuO coatings to daylight.

A similar relationship can be observed in the case of exposure of membranes to daylight (Figure 8). Similar to the case of irradiation with UV light, the factor determining photocatalytic properties under the influence of visible light is the magnetron power of the copper. This conclusion can be confirmed by comparative tests of photocatalytic properties of multi-component TiO_2_ + CuO coatings and Ti and Cu coatings (Figure 9). It was observed that even though the TiO_2_ coating does not have photocatalytic properties in visible light, the multi-component TiO_2_ + CuO coating showed a dye reduction comparable to the dye reduction of the one-component Cu coating.

In the case of coatings deposited at the magnetron powers P_M-Cu_ 100 W and 200 W, an absorbance of methylene blue dye in visible light was reduced by approx. 40% compared to the non-coated membrane (Figure 8). It was also noticed that the TiO_2_ + CuO coatings deposited at the P_M-Cu_ = 15 W magnetron power do not show photocatalytic properties both in UV light and in visible light. This is probably due to the insufficient thickness of the deposited coating, which is caused by the low magnetron power P_M-Cu_ and the low deposition rate of copper particles.

### 3.4. Surface Free Energy

**Figure 10:**
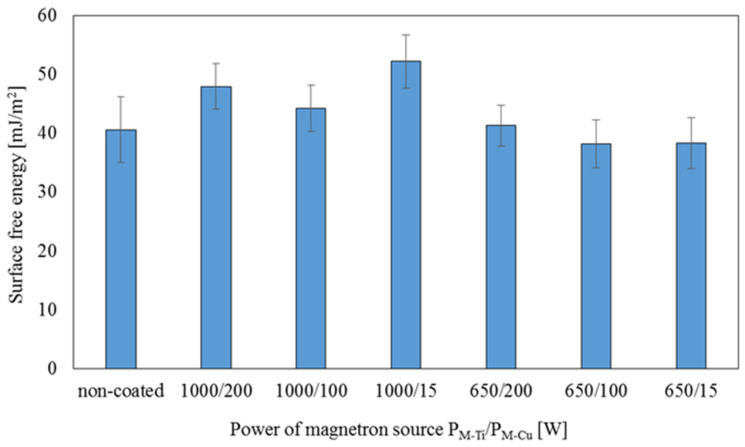
Comparison of the surface free energy for non-coated membrane and membranes covered with TiO_2_ + CuO coatings deposited within 30 s at different magnetron power (P_M-Ti_/P_M-Cu_).

The surface free energy was calculated for each tested coating based on the contact angles for deionized water and diodomethane. The results are presented in Table 2 and Figure 10.

The obtained results indicate that the contact angle of the polar liquid decreases with the increase in the magnetron power of Ti. For coatings deposited at the magnetron power of P_M-Ti_ = 1000 W, a decrease in the wetting angle was observed on average by approx. 36% compared to the non-coated membrane. On the other hand, in the case of coatings deposited at the magnetron power P_M-Ti_ = 650 W, the decrease was on average approx. 15%. However, no significant differences were observed between the values of contact angles for a non-polar liquid (diodomethane) for coatings deposited at the magnetron power P_M-Ti_ = 650 W—max. ± 8%.

The surface free energy of the membranes with deposited TiO_2_ + CuO coatings was based on the polar and dispersion components. The coatings deposited at the magnetron power P_M-Ti_ = 1000 W have observed an increase in SFE value, which proves the increase in hydrophilicity of these materials. According to the literature data, hydrophilic materials promote the growth of bacteria through the increased adhesion of microorganisms to the substrate, and thus the possibility of their multiplication. However, titanium dioxide (TiO_2_) doped with copper oxide (CuO) has a beneficial effect on the functional properties of the tested membranes. Due to the presence of titanium dioxide, which causes an increase in hydrophilicity, the filtration properties of the membranes should not be disturbed. Copper oxide (CuO), which has bactericidal properties, inhibits the growth of microorganisms preventing biofouling formation.

### 3.5. Filtration and Separation Performance

**Figure 11:**
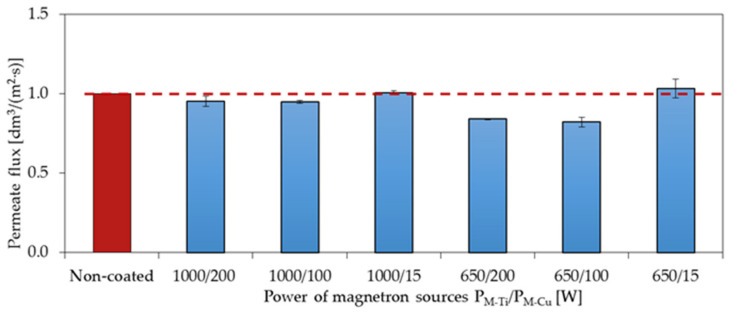
The effect of changing the magnetron power P_M-Ti_ and P_M-Cu_ in the deposition process of the TiO_2_ + CuO coatings on the membrane surface on the permeate flux determined during filtration.

The membranes with deposited TiO_2_ + CuO coatings were also tested in terms of their filtration and separation properties. The filtration properties were measured by the determined volumetric permeate flux calculated through the time needed to filter 100 mL of H_2_O (Figure 11).

The obtained values of the permeate flux for the coatings deposited at the magnetron power P_M-Ti_ = 1000 W are similar to the values obtained for the non-coated membrane. For the coatings deposited at the magnetron powers P_M-Ti_ = 650 W and P_M-Cu_ = 100 and 200 W, a decrease in the value of 16% and 18% was observed, respectively.

**Figure 12:**
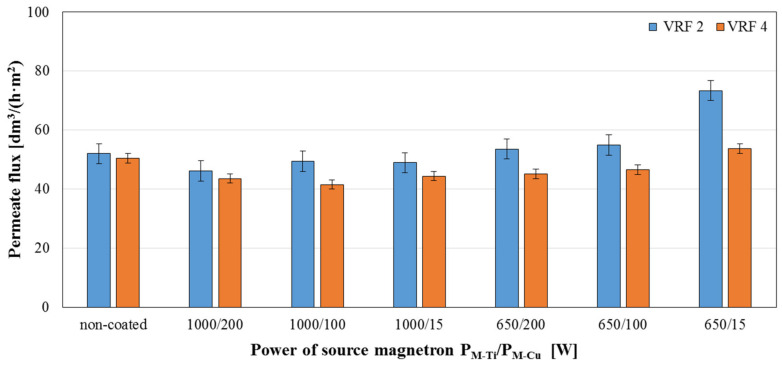
Permeate fluxes determined for 2-fold (VRF 2) and 4-fold (VRF 4) reduction in the volume of the model dairy wastewater in the filtration processes using a non-coated membrane and membranes with deposited TiO_2_ + CuO coatings.

**Figure 13:**
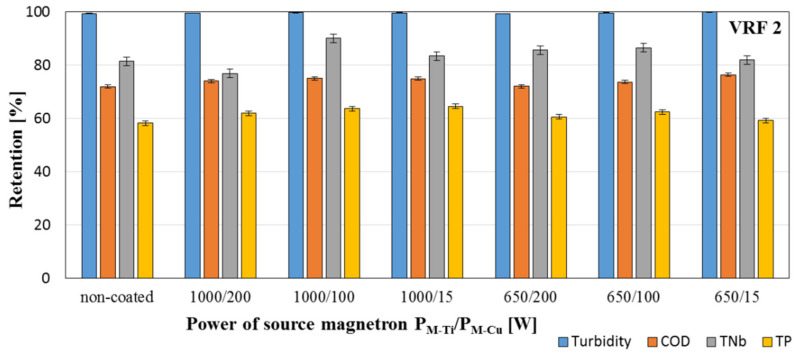
Retention coefficients of individual parameters of model dairy wastewater obtained after a 2-fold reduction in the volume of the feed in the filtration process using a non-coated membrane and membranes with deposited TiO_2_ + CuO coatings.

**Figure 14:**
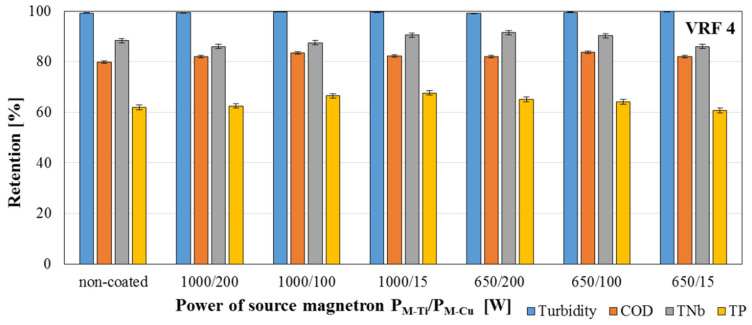
Retention coefficients of individual parameters of model dairy wastewater obtained after a 4-fold reduction in the volume of the feed in the filtration process using a non-coated membrane and membranes with deposited TiO_2_ + CuO coatings.

The separation properties were checked by the model dairy wastewater treatment process was carried out determining the retention degree of individual parameters and the volumetric permeate flux determined through the time needed to reduce the volume of the feed twice (VRF 2) and four (VRF 4) times. The obtained results are shown in Figure 12, Figure 13 and Figure 14.

Based on obtained Figure 12, Figure 13 and Figure 14, it was noticed that using the magnetron powers P_M-Ti_ = 650 W and P_M Cu_ = 15 W for deposition of presented coatings resulted in an increase in the permeate flux value by 40% for VRF = 2 and 15% for VRF = 4. No significant differences were observed in the values of the permeate fluxes, and thus no changes in the filtration properties of the modified membranes compared to the non-coated membrane (Figure 12). The values of the retention coefficients obtained after the two2- and four-fold reduction of the feed volume in the filtration process of the model dairy sewage showed that the deposition of two-component TiO_2_ + CuO coatings on polymer membranes does not disturb the separation properties of these membranes. For most of the tested coatings, a slight increase was observed in the retention coefficients of individual parameters. Among the tested coatings, the highest values of retention coefficients were characteristic for the membrane with TiO_2_ + CuO coating deposited at the magnetron power P_M-Ti_ = 1000 W and P_M-Cu_ = 15 W—99.6% for turbidity, 82.3% for COD, 90.6% for TNb, and 67.7% for TP.

## 4. Conclusions

In this work, the polyamide membranes were coated with two-component TiO_2_ + CuO coatings. The article presents the effect of the magnetron power of multi-component TiO_2_ + CuO deposition on the surface of polymer membranes on their functional properties. The analysis of the surface of the membranes surface with the TiO_2_ + CuO coating produced at the magnetron power P_M-Cu_ = 15 W showed no changes in the porous structure of the membrane. The higher magnetron powers (P_M-Cu_ = 100 W or P_M-Cu_ = 200 W) resulted in an increase in the proportion and diameter of pores in the membrane structure. However, these changes did not affect the permeability of the membrane. The results of the tests on bactericidal properties showed a significant impact of the magnetron power on the biocidal effectiveness of the obtained membranes. The highest antibacterial activity against both bacteria was found in TiO_2_ + CuO coatings obtained with magnetron powers: P_M-Ti_ = 1000 W, P_M-Cu_ = 200 W and P_M-Ti_ = 650 W, P_M-Cu_ = 200 W. Chemical composition analysis showed that the coatings characterized by the higher Cu content contribute to stronger antibacterial efficiency. The conducted research has also shown that membranes with TiO_2_ + CuO coatings have very good photocatalytic properties under UV radiation. In the case of TiO_2_ + CuO coatings produced at the power of P_M Cu_ = 100 W and 200 W, we observed a 90% reduction of the dye after 24 h of irradiation. Only in the case of TiO_2_ + CuO coatings obtained with the magnetron power P_M_-_Cu_ = 15 W, only about 20% reduction of the dye was observed. This is probably due to insufficient coating thickness. At the same time, the authors did not find any negative impact of the coatings on the filtration and separation properties of the membranes. The conducted analysis of both, the retention coefficients and the volumetric permeate flux for the filtration of the model dairy wastewater, did not show significant differences for the membranes coated with TiO_2_ + CuO compared to the native(non-coated) membrane.

The presented results confirm the possibility of using thin functional coatings to modify the functional properties of polymer membranes. A thin TiO_2_ + CuO coating makes the membranes hydrophilic, which no reduces the flow of the filtered medium through the membrane and no reducing the speed of the filtration process In addition, the obtained bactericidal properties can reduce the intensity of microbial multiplication and biofilm formation on the membrane surface preventing biological contamination of the filtered medium. The photocatalytic properties of the membranes may be useful for a process that is carried out by accelerating chemical transformations, intensifying the decomposition and transformations of substances in the system, and neutralizing impurities from the filtration process. The obtained results are very promising for the potential application of TiO_2_ + CuO coatings produced by magnetron technology to reduce the biofouling phenomenon in the membrane filtration process.

## Figures and Tables

**Table 1:** Deposition parameters for TiO_2_ + CuO coatings.

Power of Ti Magnetron Source [W]	Power of Cu Magnetron Source [W]	Atmosphere	Pressure [mbar]	Time of the Process [s]
650	15	10% O_2_ + 90% Ar	5.0 × 10^−3^	30
650	100	10% O_2_ + 90% Ar	5.0 × 10^−3^	30
650	200	10% O_2_ + 90% Ar	5.0 × 10^−3^	30
1000	15	10% O_2_ + 90% Ar	5.0 × 10^−3^	30
1000	100	10% O_2_ + 90% Ar	5.0 × 10^−3^	30
1000	200	10% O_2_ + 90% Ar	5.0 × 10^−3^	30

**Table 2:** Contact angles of the tested TiO_2_ + CuO coatings.

Samples	Contact Angle [°]	
	Water	Diodomethane
Non-coated	98.8 ± 2.4	38.8 ± 5.0
650 W Ti/15 W Cu	85.1 ± 3.1	40.8 ± 3.1
650 W Ti/100 W Cu	85.5 ± 2.5	41.9 ± 3.3
650 W Ti/200 W Cu	80.4 ± 2.2	36.4 ± 2.8
1000 W Ti/15 W Cu	56.2 ± 3.6	31.3 ± 2.8
1000 W Ti/100 W Cu	68.2 ± 2.5	37.2 ± 3.1
1000 W Ti/200 W Cu	63.4 ± 2.3	31.4 ± 3.1

## Data Availability

Not applicable.
